# The Effect of Nitriding Temperature of AISI 316L Steel on Sub-Zero Corrosion Resistance in C_2_H_5_OH

**DOI:** 10.3390/ma17133056

**Published:** 2024-06-21

**Authors:** Beata Kucharska, Janusz Kamiński, Krzysztof Kulikowski, Tomasz Borowski, Jerzy Robert Sobiecki, Tadeusz Wierzchoń

**Affiliations:** Faculty of Materials Science and Engineering, Warsaw University of Technology, ul. Wołoska 141, 02-507 Warsaw, Poland; janusz.kaminski@pw.edu.pl (J.K.); krzysztof.kulikowski@pw.edu.pl (K.K.); tomasz.borowski@pw.edu.pl (T.B.); jerzy.sobiecki@pw.edu.pl (J.R.S.); tadeusz.wierzchon@pw.edu.pl (T.W.)

**Keywords:** austenitic steel, plasma nitriding, corrosion resistance, ethanol, contact angle

## Abstract

In this paper, glow nitriding processes at cathode potential are used at various temperatures to investigate how they affect the corrosion resistance of 316L steel in ethanol at temperatures of 22 °C and −30 °C. Lowering the test temperature reduces the corrosion rate of the nitrided layers. Conversely, glow nitriding at 450 °C improves the corrosion resistance of the tested steel. Increasing the nitriding temperature to 520 °C increases the corrosion rate. It should be noted that the ethyl alcohol solution, due to the lack of aggressive ions, does not cause significant changes in the corrosion rate of the steel. The value of the corrosion current varies in the range of 10^−2^–10^−3^ µA/cm^2^. Nitrided layers increase the contact angle measured for water and are entirely wettable for ethanol. The objective of this study is to evaluate the effect of the nitriding temperature of AISI 316L steel on its corrosion resistance in an ethanol solution at room temperature and at −30 °C.

## 1. Introduction

Austenitic stainless steels (ASSs) are of great interest due to their excellent formability, corrosion resistance, and biocompatibility [1,2]. ASSs are widely used in the chemical, oil and gas, and orthopedic industries due to their high resistance to corrosion processes, which is mainly due to their high chromium content, sometimes with the addition of molybdenum in solid solution, which is responsible for the formation of a natural self-healing passive layer [3]. However, their low hardness and low resistance to frictional wear severely limit their industrial application. An increasing number of thermochemical methods have been used to improve the surface of ASSs, including nitriding [4,5,6,7,8,9,10], carburizing [9,11,12,13,14], nitrocarburizing [13,15,16], oxynitriding [17], and boriding [18]. Of the above technologies, the most widely used and researched is glow discharge nitriding, which, at a low nitriding temperature (below 450 °C) and a short process time (less than 6 h), produces a precipitation-free nitrogen austenite (γN) on the surface, the so-called S phase (expanded austenite), with high corrosion resistance and good wear resistance [15,16,19]. Exceeding this nitriding temperature can lead to the formation of CrN precipitates, resulting in chromium-depleted zones [6,20]. Higher process temperatures, due to the additional precipitation of cubic (γ′-Fe_4_N) and hexagonal (ε-Fe_2–3_N) nitrides, improve wear resistance but significantly reduce the corrosion resistance of ASSs in the presence of chlorides [21,22]. Therefore, the nitriding temperature is a critical factor affecting the corrosion resistance of the nitrided layer. The centrifugal medical cannabis extractor developed in this project, which is mainly made of ASSs, operates at −30 °C in an ethanol solution. There is a large body of work investigating the corrosion resistance of nitrided ASSs, particularly in chloride-containing environments [23,24], which have the greatest influence on the pitting of the ASS surface. Tests are mostly conducted at room temperature, but also at 37 °C, a temperature comparable to that of the human body, when testing in solutions simulating human body fluids [25]. Fewer studies are conducted in oxidizing solutions, such as acids [26,27] and alkalis [28]. However, it has been noted that there is an absolute lack of literature data on the corrosion resistance of ASSs in alcohols, and in particular in C_2_H_5_OH ethanol.

The aim of this study was to evaluate the effect of the nitriding temperature of AISI 316L steel on its corrosion resistance, measured by potentiodynamic method and impedance spectroscopy in an ethanol solution at room temperature and at −30 °C, as well as on the contact angle of the tested surfaces, also measured using ethanol.

## 2. Materials and Methods

### 2.1. Substrate and Layer Deposition

AISI 316L type 17/10/2 alloy steel (Cr/Ni/Mo) was used as the substrate. The specimens were cut from bars with a diameter of Φ 14 and then ground on sandpaper up to 1200′. The glow nitriding processes were carried out at the cathode potential (the specimen was negatively polarized) for 6 h under the 2 mbar pressure in N_2_/H_2_ atmosphere (1/1 ratio). The glow nitriding processes were carried out at variable temperatures (450 °C, 480 °C, and 520 °C) with the other technological parameters remaining constant.

In the case of each process, cathodic sputtering was carried out at a temperature of 430 °C in an Ar/H_2_/N_2_ = 2/2/1 atmosphere for 30 min while the processed specimens were being heated, in order to clean and activate the specimen surfaces and increase the number of structural defects in the near-surface zone, leading to the formation of a nitrided layer of greater thickness as a result of accelerated diffusion processes.

### 2.2. Microstructure, Morphology, and Chemical Composition Studies

The specimens were cut on a precision saw and then hot-incorporated in conductive phenolic resin with graphite filler. Their cross-sections were then ground with 240–1200 grit sandpaper and polished with an Al_2_O_3_ suspension. The layer structure was revealed by etching the specimens with a reagent consisting of 50% HCl + 25% HNO_3_ + 25% H_2_O. The microstructure, surface morphology, and chemical composition of the layers were examined using a scanning electron microscope (S3500N, HITACHI, Tokyo, Japan) equipped with the energy-dispersive spectroscopy (EDS) microanalysis system.

### 2.3. XRD Studies

The XRD studies of the nitrided layers were performed on an X-ray diffractometer (D8 ADVANCE, Bruker, Billerica, MA, USA) using filtered Cu Kα radiation (λ = 0.154056 nm) at room temperature. The conditions were as follows: voltage—40 kV, current—40 mA, angular range 2θ—from 15° to 120°, step Δ2Θ—0.05°, and counting time—3 s.

### 2.4. Microhardness Tests

Microhardness tests were carried out on a hardness tester (HMV-G, Shimadzu, Kyoto, Japan) using the Vickers method using loads of 0.2 and 0.5 kg.

### 2.5. Corrosion Tests

**Figure 1:**
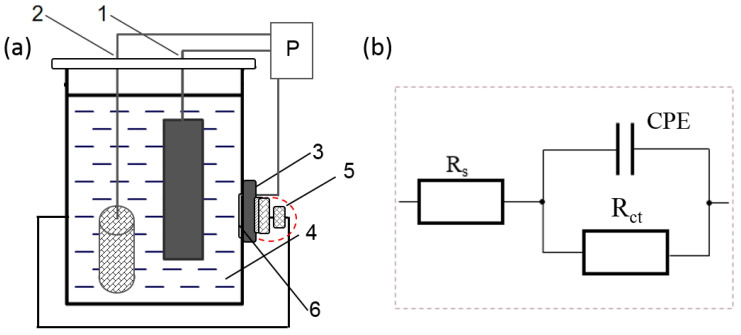
(**a**) Scheme of an electrochemical cell for corrosion resistance studies: 1—reference electrode, 2—auxiliary electrode (platinum electrode), 3—test electrode (specimen), 4—99% ethanol environment, P—potentiostat, 5—insulator with a spring and a screw pressing the sample to the corrosion cell, and 6—hole in the vessel (20 mm); (**b**) model of the equivalent circuit proposed for curve fitting of the EIS data.

Corrosion resistance tests were carried out using the electrochemical impedance spectroscopy (EIS) and potentiodynamic methods in 99% C_2_H_5_OH water (1%) solution using a potentiostat (AutoLab PGSTAT100, Atlas-Sollich, Kartuzy County, Poland) at −30 °C and 22 °C. The choice of a low test temperature and ethanol for a corrosive environment was due to the potential use of this material to construct an extractor using ethanol operating at low temperatures. The tests were carried out in a three-electrode system: test electrode, reference electrode (the silver/silver chloride SSC electrode), and auxiliary electrode (platinum mesh: total height 150 mm, cylinder diameter 35 mm) (Figure 1a). The application of silver electrodes (118 mV vs. RHEK—Reversible Hydrogen Electrode kit (25 °C)) made it possible to conduct electrochemical tests both in anhydrous environments and at reduced temperatures.

#### 2.5.1. Electrochemical Impedance Spectroscopy Method

Impedance tests were carried out in the frequency range 10^5^–10^−3^ Hz, with a sinusoidal signal amplitude of 20 mV, at an open-circuit potential (OCP). The impedance spectra were analyzed using Baukamp’s EQUIVCRT program (version 4.9.007). Prior to the tests, the specimens were stabilized in a currentless condition to determine the open system potential (OCP) for a period of 10,000 s. An electrical equivalent system (Figure 1b) with two time constants was used to analyze the obtained spectra, allowing the characterization of both the high resistance of the electrolyte (99% C_2_H_5_OH) and the properties of the double layer formed at the metal–electrolyte interface. R_s_ denotes the electrolyte resistance, Y0-CPE is the constant phase element (CPE) at the interface between the electrolyte and specimen, and R_ct_ is the charge transfer resistance of the specimens. The selection of the electrical equivalent system for the obtained impedance spectra was conditional on both the image of the corrosion damage and on the smallest matching errors of the system elements (determined using the least-square method). The obtained spectra were presented in the form of Bode and Nyquist plots.

#### 2.5.2. Polarization Method

Polarization resistance was tested using the Stern method by polarizing the test material from a potential 10 mV below to 10 mV above the determined open-circuit potential at a sweep rate of 0.2 mV/s. The polarization resistance (Rpol) was determined from the E = f(i) dependence. Potentiodynamic tests were performed at a potential of 3000 mV. The materials were polarized at a potential change rate of 0.2 mV/s. The corrosion current density (I_corr_) and corrosion potential (E_corr_) were determined using the Tafel extrapolation method.

### 2.6. Contact Angle Measurements

Contact angle tests were carried out using a goniometer (90-U3-PRO, RAME-HART, Ledgewood, NJ, USA) equipped with a CCD (charge-coupled device) camera, an adjustable stage, a syringe for the manual dosing of drops, and DROPImage software (ramé-hart DROPimage Pro). The liquid used in the study was distilled water and a 99% C_2_H_5_OH solution. A total of five measurements were performed on each specimen.

## 3. Results and Discussion

### 3.1. Effect of Nitriding Temperature on the Surface Morphology, Microstructure, and Chemical Composition

**Figure 2:**
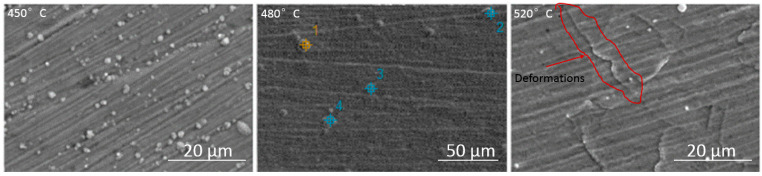
Morphology and topography of the surface of nitrided layers at temperatures of 450 °C, 480 °C, and 520 °C.

Small deposits on the surface of the layers (Figure 2) were formed as a result of the cathodic sputtering of the steel table on which the specimens were placed (Table 1). No other elements indicating contamination were observed. Deformations were identified near the grain boundaries, which were caused by significant stresses generated during the diffusion supersaturation of grains with different crystallographic orientations. The nitriding process led to a varying degree of expansion of adjacent grains and created a relief visible on the steel surface. The higher nitriding temperature resulted in the greatest saturation of the layer and relief effect. The surface of the layer produced in these conditions was the most cracked (Figure 2) [29,30].

**Figure 3:**
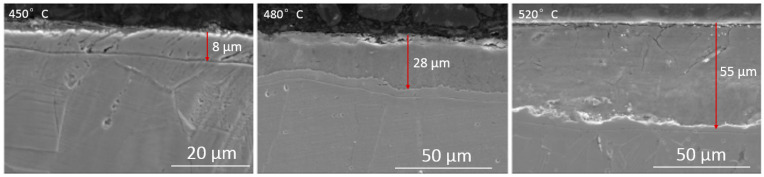
Cross-sections of the layers nitrided at temperatures of 450 °C, 480 °C, and 520 °C.

The layers were characterized by different thicknesses depending on the process temperature (Figure 3). The nitrided layer produced at 450 °C was about 8 µm thick, while at 480 °C, it was about 28 µm thick, and at 520 °C, it was about 60 µm thick. The layers nitrided at 480 and 520 °C show a double structure with a thinner sublayer closer to the core. This is a layer of carbon austenite formed as a result of the movement of carbon contained in the steel into deeper zones of the layer through the action of nitrogen (Figure 3).

### 3.2. XRD Studies

**Figure 4:**
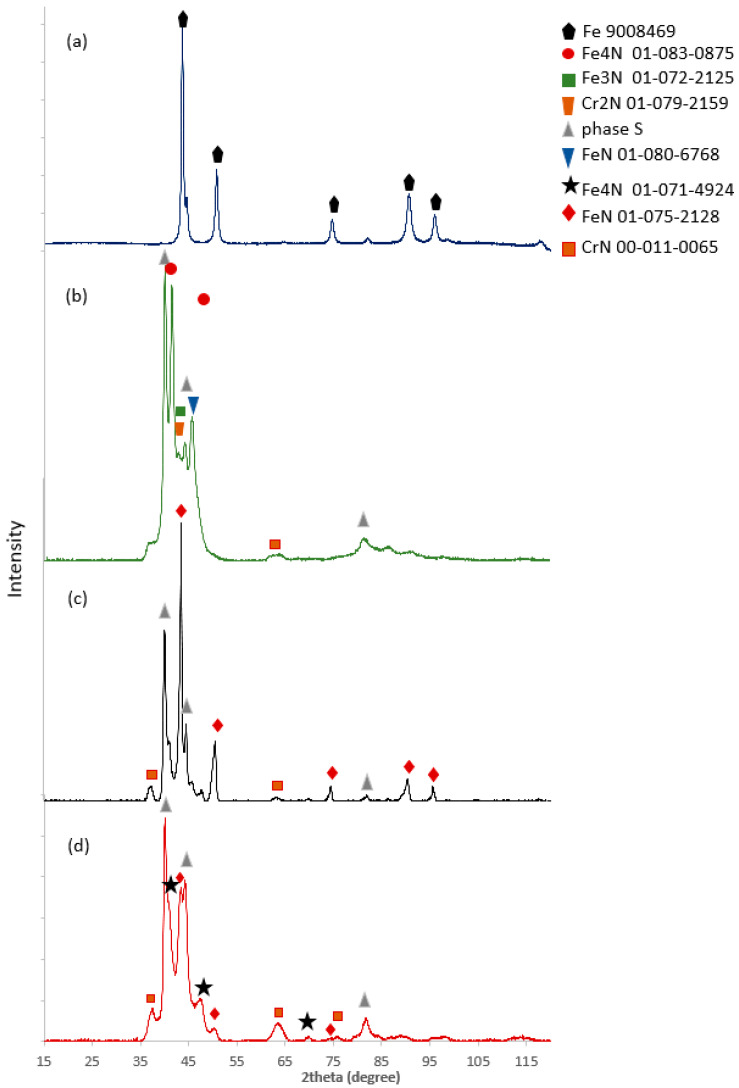
XRD results of (**a**) 316L substrate and the layers nitrided at temperatures of (**b**) 450 °C, (**c**) 480 °C, and (**d**) 520 °C studied on the surface.

Figure 4 shows the phase diffractograms of the tested specimens with nitrided layers. The 316L steel substrate was composed of the austenitic phase, and no other phases were detected in the substrate (Figure 4). In the case of the nitrided layer at a temperature of 450 °C, the S phase (nitrogen austenite), iron nitride Fe_4_N, and trace amounts of chromium nitride CrN were identified. The S phase and the Fe_4_N nitride showed the highest peak intensity. In turn, in the layer formed at a temperature of 480 °C, nitrogen austenite and iron nitrides Fe_4_N and FeN were also observed. Signals originating from CrN were characterized by the lowest intensity. In the case of the layer produced at 520 °C, the peaks from the S phase and the Fe_4_N nitride showed the highest intensity. The peaks from chromium nitride CrN were also the most intense compared to the layers produced at a lower temperature.

### 3.3. Microhardness

The microhardness of 316L steel in its initial state was approximately 336 HV0.2 (Table 2). As a result of the glow nitriding process, the surface microhardness increased to approximately 796 HV0.2 and 714 HV0.2 for the 450 °C and 480 °C processes, and to approximately 1412 HV0.2 for the 520 °C process. The increase in hardness was the result of the formation of an “s” zone on the surface, approximately 4 μm thick, and an outer zone of FeN and Fe_4_N iron nitrides (Figure 3), the thickness of which increased with the process temperature. In the case of the layer produced at a temperature of 520 °C, the surface hardness obtained during measurements under a load of 0.2 kg is related to its large thickness (approximately 60 μm), which allows the unhardened substrate to participate to a small extent in the plastic deformation process. The layers produced at temperatures of 450 °C and 480 °C are characterized by a much smaller thickness of the surface nitride zone (Figure 3), and when tested under high loads (0.2 kg and 0.5 kg), the influence of the plastic deformation of the substrate was clearly visible. A significant influence of the load size on the microhardness value was also observed. This is due to the fact that in the case of lower loads, the substrate had no influence on the microhardness values. In the case of increasing the load by 2.5 times, a clear influence of the substrate on the hardness was observed, which is related to the depth of penetration of the indenter during the test.

### 3.4. Corrosion Studies

**Figure 5:**
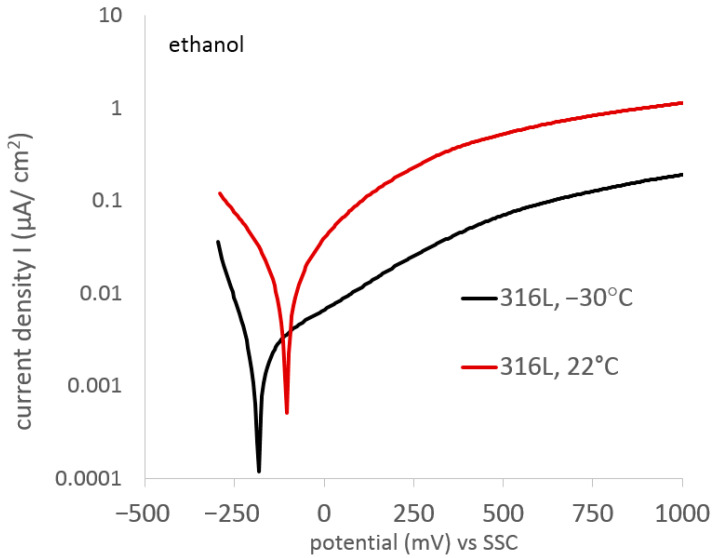
Polarization curves of 316L steel in its initial state exposed to 99% C_2_H_5_OH at temperatures of 22° and −30 °C.

Figure 5 shows the polarization curves of AISI 316L steel in its initial state, exposed to temperatures of −30 °C and 22 °C. The analysis of the curves shows an increase in the corrosion resistance of the steel as the exposure temperature decreases, as well as a slight shift in the corrosion potentials toward the cathodic side as the temperature decreases. The reduction in the electrochemical activity of electrode processes is determined using the Arrhenius equation ($k=Ae^{\frac{{-E}_{a}}{RT}}$, *k*—rate constant, *A*—pre-expotential factor, *E_a_*—activation energy, *R*—universal gas constant, and *T*—absolute temperature), which expresses the dependence of the reaction rate constant on temperature.

Table 3 summarizes the characteristic electrochemical values obtained from potentiodynamic tests.

**Figure 6:**
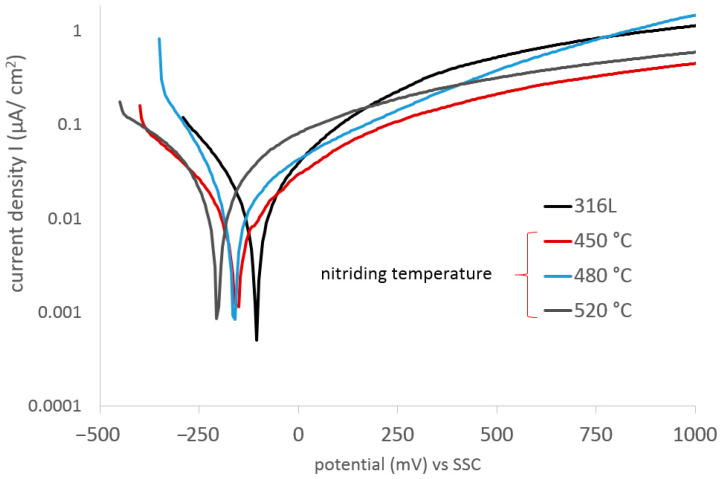
Potentiodynamic curves of 316L steel and nitrided 316L steel, exposed in ethyl alcohol at 22 °C.

The applied glow nitriding processes at the cathode potential, due to the high electrochemical resistance (~1 M Ωcm^2^) and the low aggressiveness of the corrosive environment (lack of chloride ions), did not significantly affect the changes in the corrosion resistance and the durability of the nitrided layers produced (Figure 6). However, the influence of increasing the nitriding temperature on the corrosion current density was observed (correlation coefficient = 0.92). No distortions were observed during potentiodynamic studies despite using a potential scan rate of 0.2 mV/s [31].

**Figure 7:**
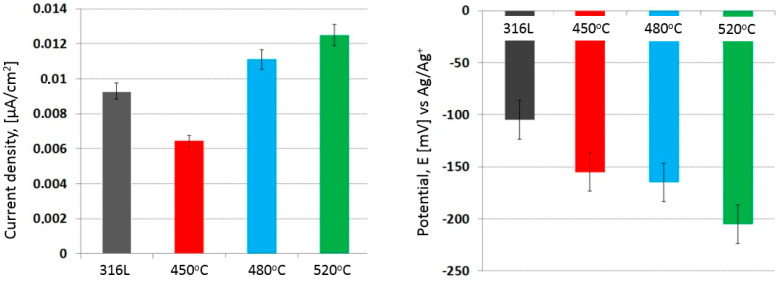
Characteristic electrochemical values of 316L steel and nitrided 316L steel exposed in ethyl alcohol at 22 °C.

It can be noticed that the produced layers are anodic in relation to the substrate (Figure 6 and Figure 7; Table 4), which could favor the etching of the layers as a result of long-term exposure. However, due to the high solubility of oxygen in ethyl alcohol ($C_{O_{2}}\;$7.5–11.6 mM (25 °C)) [32], any defects in the layer (as well as nondefected areas) have the potential to auto-oxidize and form oxy-nitrides, which, in the absence of chlorides in the solution, would have a positive effect on both the corrosion resistance and the durability of the surface layers.

The shape of the potentiodynamic curves indicates the lack of pitting corrosion in the entire range of the tested potentials. The observed constant increase in the current density value with increasing polarization intensity indicates the low-intensity uniform corrosion of the nitrided layer; current densities in the surface layer (at 2000 mV) were approximately 1 µA/cm^2^ in the case of low-temperature glow nitriding and approximately 5 µA/cm^2^ in the case of the layer produced at 520 °C. This result indicates a slightly increased intensity of electrode processes taking place on the tested surface, probably due to the increased roughness of the substrate.

A similar tendency was also observed in the case of potentiodynamic tests at a lower (−30 °C) temperature (correlation coefficient = 0.93). However, due to the reduction in the intensity of electrochemical processes (according to the Arrhenius equation), the corrosion current densities were an order of magnitude lower than those of the tests at room temperature.

**Figure 8:**
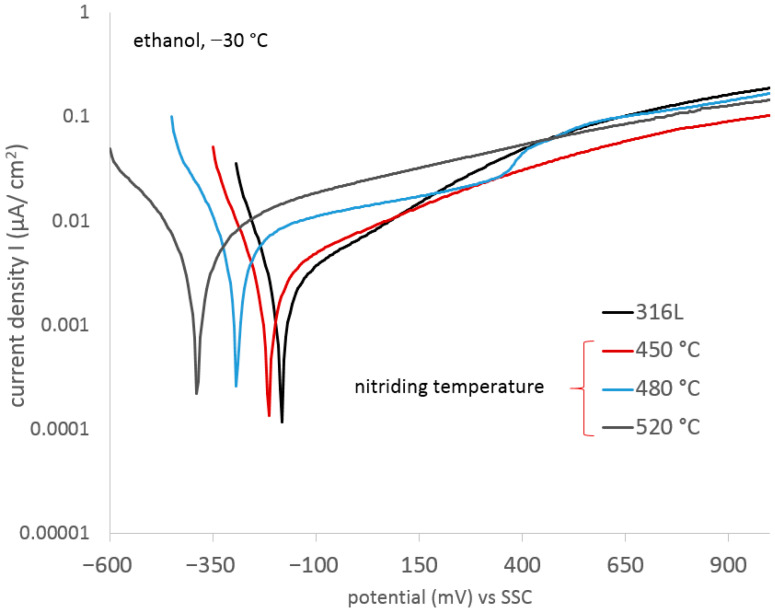
Potentiodynamic curves of 316L steel and nitrided 316L steel (A450, A480, and A520), exposed in ethyl alcohol at −30 °C.

**Figure 9:**
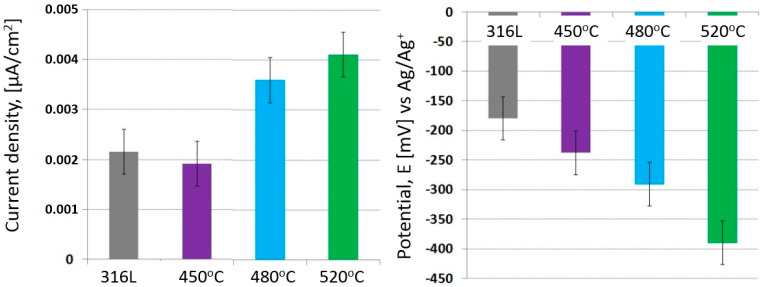
Characteristic electrochemical values of 316L steel and nitrided 316L steel exposed in ethyl alcohol at −30 °C.

Additionally, an additional electrochemical process was observed (at a potential of approx. 400 mV (Figure 8)), increasing the intensity of anodic processes. It can be assumed that the additional electrochemical process is related to the oxidation of the nitrided layer with the simultaneous formation of oxygen and nitrides on the surface. However, due to the lack of chloride ions in the solution, pitting corrosion initiation was not observed in these areas. The best corrosion resistance occurred in the case of layer nitride at 450 °C (Figure 9, Table 5).

**Figure 10:**
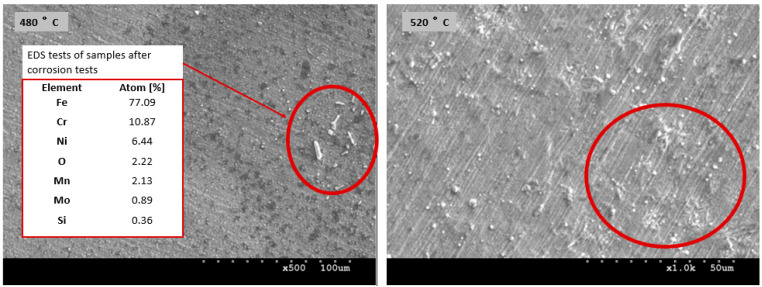
Surfaces of layers nitrided at temperatures of 480 °C and 520 °C after corrosion tests in ethyl alcohol (22 °C) with corrosion products (red circles) and chemical composition of corrosion products.

Figure 10 shows the surfaces of layers nitrided at temperatures of 480 °C and 520 °C after corrosion tests in an ethyl alcohol (22 °C). In both cases, the local etching of the nitrided layer was observed, producing dielectric oxides.

**Figure 11:**
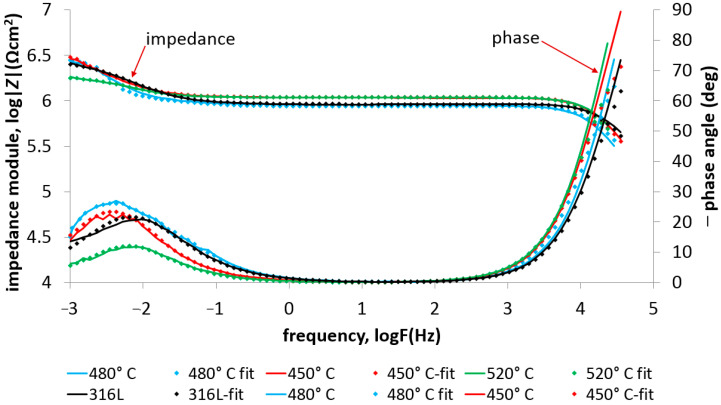
EIS spectra with fitted data of 316L steel with nitrided layers exposed in 99% C_2_H_5_OH at 22 °C.

**Figure 12:**
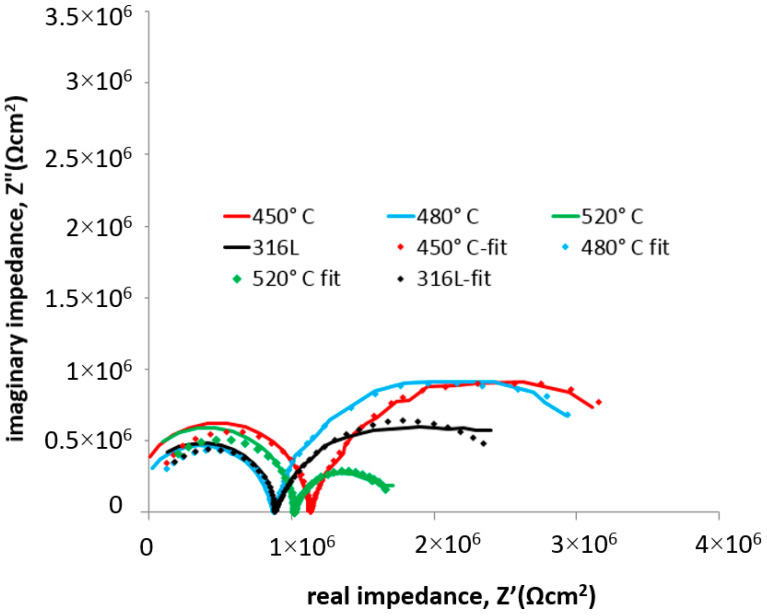
Nyquist spectra with fitted data of 316L steel with nitrided layers exposed in 99% C_2_H_5_OH at 22 °C.

**Figure 13:**
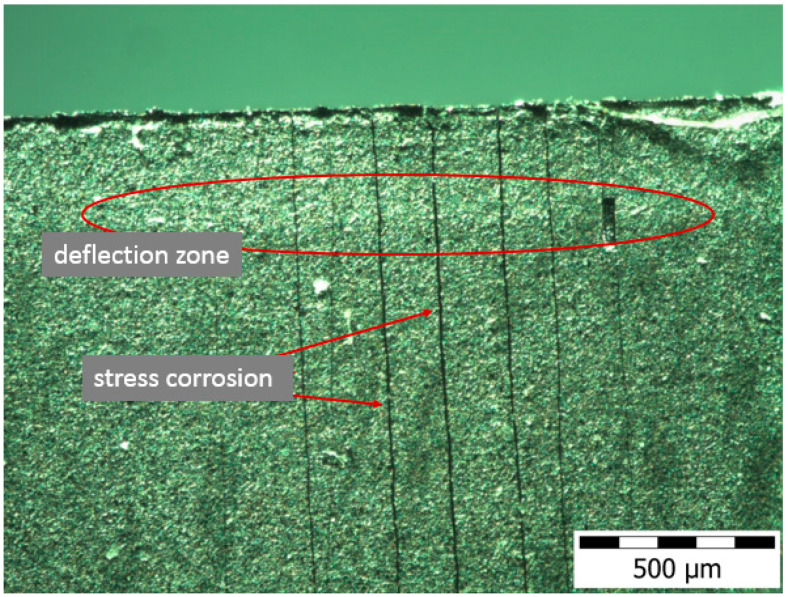
SEM studies of the surface of stress corrosion of layer nitrided at 520 °C as a result of long-term (3 days) exposure at −30 °C (on bent specimen).

The impedance test results (Figure 11 and Figure 12) confirmed the positive effect of low-temperature (450–480 °C) glow nitriding on the corrosion resistance of 316L steel. Increasing the glow treatment temperature (520 °C) resulted in a significant reduction in corrosion resistance (Table 6) due to both a significant increase in surface development and the possibility of chromium nitride (CrN) formation at the grain boundaries, which on the one hand increases hardness but may also initiate intergranular corrosion. This is confirmed by the work of Skolek et al., which shows the possibility of chromium nitride formation at temperatures above 440 °C [33]. The observed increase in layer hardness also led to stress corrosion cracking in the deflection zone of the specimen as a result of external stresses and long-term exposure at −30 °C (Figure 13).

**Figure 14:**
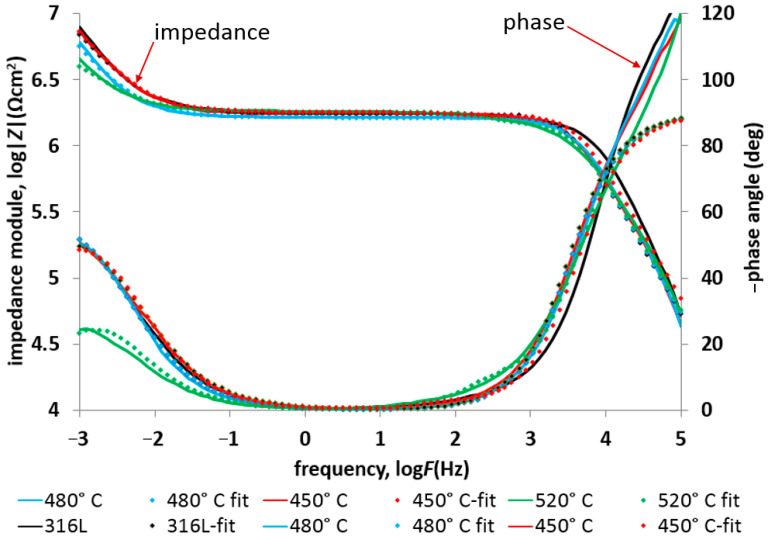
EIS spectra with fitted data of 316L steel with nitrided layers exposed in 99% C_2_H_5_OH at −30 °C s.

**Figure 15:**
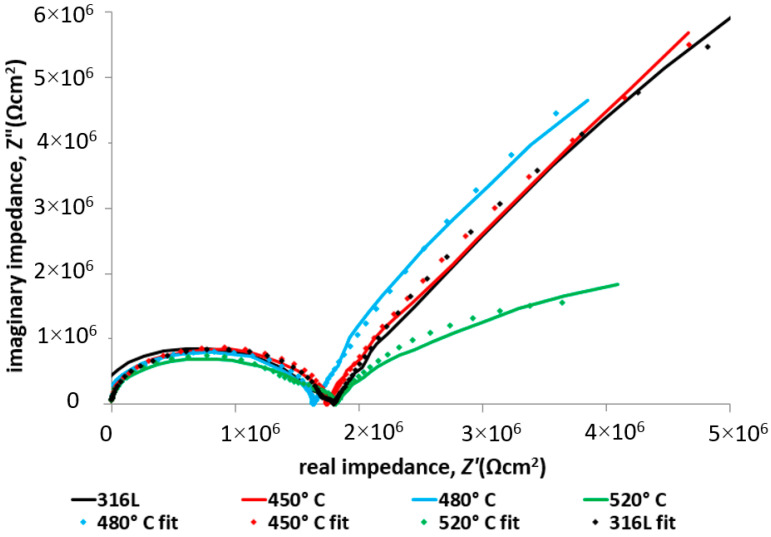
Nyquist spectra with fitted data of 316L steel with nitrided layers exposed in 99% C_2_H_5_OH at −30 °C.

Figure 14 and Figure 15 show the impedance spectra (Bode and Nyquist, respectively) of the tested layers compared to 316L exposed at lower (−30 °C) temperature, exposed in 99% C_2_H_5_OH, while Table 7 lists the characteristic electrochemical values of the double layer present at the nitrided layer–electrolyte phase boundary.

The observed high-frequency peaks in the impedance spectra characterize the resistivity of the environment. In the case of the 316L steel, the resistance of the environment at room temperature was significant and amounted to approximately 0.79 × 10^6^ Ωcm^2^. When the temperature decreased to around −30 °C, the value further increased to 2.01 × 10^6^ Ωcm^2^. The increase in solution resistance as a result of lowering the temperature is one of the reasons for the observed decrease in corrosion current density in the case of potentiodynamic tests (Table 5). In the case of impedance tests, where it is possible to eliminate the influence of the resistance of the environment on the remaining electrochemical results (i.e., the resistance of charge through the double layer (resistance R_ct_) and the capacity of the double layer or the value of the ‘n’ parameter), we can precisely determine the influence of the glow treatment temperature on the corrosion resistance of nitrided layers exposed in ethyl alcohol. The analysis of the change in the resistance value R_ct_ indicates a decrease in its value in proportion to the increase in the glow treatment temperature. This trend was observed regardless of the exposure temperature (i.e., 22 °C and −30 °C). Additionally, in the impedance spectra of nitrided layers produced at elevated temperature (520 °C), a slightly marked additional capacitive peak was observed (in the frequency range 10^−3^–10^5^ Hz), which was caused by an increase in the roughness of the surface layer.

The constant ‘n’ parameter of the tested layers, amounting to approximately 0.85, indicates the passive–active nature of the surface layers exposed at a temperature of 22 °C. Slight differences in the values of the ‘n’ parameter were observed in the case of tests at a reduced temperature. The obtained variable values did not significantly change the nature of the layer; only in the case of the layer produced at 520 °C does the reduced value of the ‘n’ parameter (n = 0.67) indicate a significant influence of diffusion factors determining the electrochemical resistance of the layer. It can be assumed that the obtained result is caused by the significant development of the surface.

Figure 12 and Figure 15 show the Nyquist plots of the 316L substrate and the nitrided layers. As can be seen in the figures, in the higher temperature, there are two semicircles for each test specimen. The first semicircle is connected with electrolyte resistance. In the case of a lower temperature, only the first semicircle is clearly visible, which indicates a different corrosion mechanism. The electrolyte resistance is about twice that of a higher test temperature (Table 6 and Table 7). The higher slopes of the second semicircle at −30 degrees indicate significantly higher charge transfer resistance between the electrolyte and the tested materials under these test conditions.

### 3.5. Contact Angle

The nitrided layers were characterized by a higher water contact angle than the initial state (316L) (Table 8). The highest value of the contact angle in water was obtained for the specimen nitrided at 520 °C. Nitrided specimens were characterized by complete wettability by ethanol. 

## 4. Conclusions

1. Notably, the 99% ethyl alcohol solution was characterized by low aggressiveness due to the lack of chloride ions in the solution and low conductivity, which was further diminished by the reduced temperature. Nyquist spectra suggest different corrosion mechanisms of tested corrosion temperatures.
2. Low-temperature glow nitriding of 316L steel slightly increased the material corrosion resistance.
3. Glow nitriding at 520 °C significantly reduced the corrosion resistance of 316L steel. Additionally, due to the significant thickness and hardness of the surface layer, the material was sensitive to stress corrosion cracking.
4. The high solubility of oxygen in ethyl alcohol facilitates the oxidation of the nitrided layer.
5. Nitrided layers were characterized by a higher water contact angle than the initial state. The highest value of the contact angle in water was obtained for the specimen nitrided at 520 °C.
6. The nitrided specimens were completely wettable using ethanol.

## Figures and Tables

**Table 1:** Chemical composition of surface layer nitride at 480 °C (measurement points 1–4 marked in Figure 2).

Element	Atom [%]	SD
Fe	66.56	3.56
Cr	19.73	1.07
Ni	7.94	0.93
N	2.96	1.94
Mn	1.44	0.22
Mo	0.73	0.24
Si	0.64	0.27

**Table 2:** Microhardness of 316L steel and layers nitrided at temperatures of 450 °C, 480 °C, and 520 °C.

Specimen	HV0.2	SD	HV0.5	SD
316L steel	336	6.89	322	3.18
450 °C	796	9.15	486	19.54
480 °C	714	4.76	489	22.58
520 °C	1412	2.05	1266	17.39

**Table 3:** Characteristic electrochemical values of 316L steel.

Test Temperature	E_corr_ [mV] vs. SSC	I_corr_ [µA/cm^2^]
22 °C	−105	9.28 × 10^−3^
−30 °C	−180	2.15 × 10^−3^

**Table 4:** Characteristic electrochemical values of 316L steel and nitrided 316L steel exposed in ethyl alcohol at 22 °C.

Specimen	E_corr_ [mV] vs. SSC	I_corr_ [µA/cm^2^]
316L steel	−105	9.28 × 10^−3^
450 °C	−155	6.43 × 10^−3^
480 °C	−165	11.10 × 10^−3^
520 °C	−205	12.48 × 10^−3^

**Table 5:** Characteristic electrochemical values of 316L steel and nitrided 316L steel exposed in ethyl alcohol at −30 °C.

Specimen	E_corr_ [mV] vs. SSC	I_corr_ [µA/cm^2^]
316L steel	−180	2.15 × 10^−3^
450 °C	−215	2.09 × 10^−3^
480 °C	−290	3.60 × 10^−3^
520 °C	−390	4.10 × 10^−3^

**Table 6:** Characteristic electrochemical values of the metal–electrolyte double layer (99% C_2_H_5_OH, room temperature) after glow nitriding processes on 316L steel.

Specimen	R_s_ [MΩcm^2^]	R_ct_ [MΩcm^2^]	Error %	Y0-CPE [μF/cm^2^ s^(n−1)^]	Error %	n	Error %
316L steel	0.89	1.67	3.9	11.7	5.5	0.76	2.3
450 °C	1.12	2.54	8.7	15.2	12.2	0.83	4.2
480°C	0.88	2.25	12.6	23.6	5.4	0.85	1.8
520 °C	1.01	0.75	5.9	22.3	12.8	0.84	4.5

R_s_—environmental resistance; R_ct_—charge transfer resistance through the double layer; Y0-CPE—capacitance of the solid phase element; n—imperfection coefficient of the solid phase element (CPE); error (%)—sum of squares of deviations between measured data points and the calculation line.

**Table 7:** Characteristic electrochemical values of the metal–electrolyte double layer (99% C_2_H_5_OH, room temperature) after glow nitriding processes on 316L steel (−30 °C).

Specimen	R_s_ [MΩcm^2^]	R_ct_ [MΩcm^2^]	Error %	Y0-CPE [μF/cm^2^ s^(n−1)^]	Error %	n	Error %
316L steel	1.80	19.6	23.5	7.32	7.4	0.82	2.4
450 °C	1.73	33.1	15.4	8.55	5.1	0.80	1.7
480 °C	1.63	23.5	19.1	1.47	5.8	0.82	1.8
520 °C	1.81	4.17	8.6	5.41	8.8	0.71	2.8

R_s_—environmental resistance; R_ct_—charge transfer resistance through the double layer; Y0-CPE—capacitance of the solid phase element; n—imperfection coefficient of the solid phase element (CPE); error (%)—sum of squares of deviations between measured data points and the calculation line.

**Table 8:** The contact angle of 316L steel and coatings nitrided at 450 °C, 480 °C, and 520 °C temperature.

Specimen	Contact Angle Distilled Water [°]	SD	Contact Angle 99% Ethanol [°]	SD
316L steel	65.4	2.32	12	1.11
450 °C	85.0	2.63	Total wettability	—
480 °C	84.2	2.85	Total wettability	—
520 °C	96.3	1.02	Total wettability	—

## Data Availability

The original contributions presented in the study are included in the article, further inquiries can be directed to the corresponding author.
